# Exploring *Proteus mirabilis* Methionine tRNA Synthetase Active Site: Homology Model Construction, Molecular Dynamics, Pharmacophore and Docking Validation

**DOI:** 10.3390/ph16091263

**Published:** 2023-09-06

**Authors:** Samar S. Elbaramawi, Ahmed G. Eissa, Nada A. Noureldin, Claire Simons

**Affiliations:** 1 Department of Medicinal Chemistry, Faculty of Pharmacy, Zagazig University, Zagazig 44519, Egypt; sselbaramawy@pharmacy.zu.edu.eg (S.S.E.); ageissa@pharmacy.zu.edu.eg (A.G.E.); nanoureddine@pharmacy.zu.edu.eg (N.A.N.); 2 School of Pharmacy & Pharmaceutical Sciences, Cardiff University, King Edward VII Avenue, Cardiff CF10 3NB, UK

**Keywords:** *Proteus mirabilis*, homology model, methionyl tRNA synthetase, virtual screening, urinary tract infections, molecular dynamics

## Abstract

Currently, the treatment of *Proteus mirabilis* infections is considered to be complicated as the organism has become resistant to numerous antibiotic classes. Therefore, new inhibitors should be developed, targeting bacterial molecular functions. Methionine tRNA synthetase (MetRS), a member of the aminoacyl-tRNA synthetase family, is essential for protein biosynthesis offering a promising target for novel antibiotics discovery. In the context of computer-aided drug design (CADD), the current research presents the construction and analysis of a comparative homology model for *P. mirabilis* MetRS, enabling development of novel inhibitors with greater selectivity. Molecular Operating Environment (MOE) software was used to build a homology model for *P. mirabilis* MetRS using *Escherichia coli* MetRS as a template. The model was evaluated, and the active site of the target protein predicted from its sequence using conservation analysis. Molecular dynamic simulations were performed to evaluate the stability of the modeled protein structure. In order to evaluate the predicted active site interactions, methionine (the natural substrate of MetRS) and several inhibitors of bacterial MetRS were docked into the constructed model using MOE. After validation of the model, pharmacophore-based virtual screening for a systemically prepared dataset of compounds was performed to prove the feasibility of the proposed model, identifying possible parent compounds for further development of MetRS inhibitors against *P. mirabilis*.

## 1. Introduction

*Proteus mirabilis* (*P. mirabilis*) urinary tract infections can be either symptomatic infections causing cystitis or pyelonephritis, or asymptomatic infections leading to bacteriuria. *P. mirabilis* infections are common among elderly people and patients with type II diabetes [1,2]. Moreover, *P. mirabilis* infections can cause urolithiasis. Catheterized patients infected with *P. mirabilis* are highly susceptible to the development of urolithiasis, a complication in which bladder and kidney stones obstruct the catheter and urinary tract, making treatment more difficult. Moreover, urosepsis could be developed, which is the major cause of death due to *P. mirabilis* infections. In recent studies, *P. mirabilis* was isolated from 5 to 20% of patients with a bloodstream infection and was found to be the leading cause of death in 50% of geriatric, hospitalized patients [3,4], as it can progress to potentially life-threatening urosepsis. In addition to urinary tract infections, *P. mirabilis bacteria* is a leading cause of respiratory tract, eye, nose, ear, burn and wound infections; and has been associated with neonatal meningoencephalitis and osteomyelitis [5,6].

**Figure 1:**
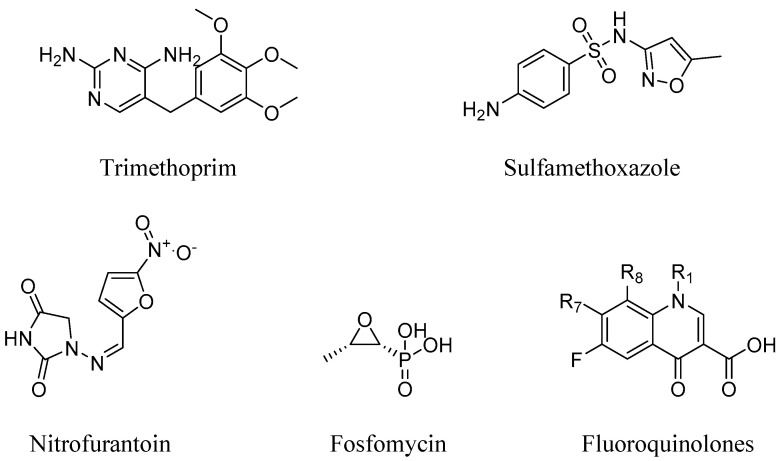
Currently used antibiotics for the treatment of *P. mirabilis*, all of which have some reported resistance.

Treatment of *P. mirabilis* infection relies on double-strength trimethoprim-sulfamethoxazole (SXT) (Figure 1) if the local SXT resistance rate is not more than 10–20% [7,8]. Recently, a wide resistance spectrum ranging from 16 to 83% of *P. mirabilis* against SXT has developed [7,8,9], and, in such cases, an alternative antibiotic therapy is recommended including fluoroquinolones, nitrofurantoin, or fosfomycin in cases of uncomplicated cystitis. However, all of these antibiotics have shown some reported resistance (Figure 1) [7,8,10].

In addition to the SXT resistance, *P. mirabilis* has developed resistance to several antibiotic classes including β-lactams, fluoroquinolones, nitrofurantoin, fosfomycin, aminoglycosides, tetracyclines, and sulfonamides [9,10,11]. *P. mirabilis* is also highly resistant to antimicrobial peptides such as polymyxin B, protegrin, LL-37, and defensin [12,13]. Aminoacyl-tRNA synthetase (AaRS) provides a potential wealth of targets in the development of drugs against *P. mirabilis* infections. The aminoacyl tRNA synthetases (AaRSs) are a group of enzymes that play an important role in protein biosynthesis. AaRSs catalyze the aminoacylation reaction of the tRNA molecule in the protein synthesis process, through two main steps. In the first step, the amino acid is activated through reaction with an ATP molecule, forming aminoacyl adenylate. In the second step, the aminoacyl adenylate reacts with its cognate tRNA molecule through esterification, and the aminoacyl tRNA is now ready for the next steps in the protein synthesis pathway. AaRSs are able to bind to and recognize all of the reactants in this aminoacylation reaction: the amino acid, ATP, and the cognate tRNA [14]. When any of these stages is inhibited, accumulation of uncharged tRNA molecules takes place, which bind to ribosomes, causing an interruption in the polypeptide chain elongation [15]. There are more than 20 types of AaRS enzymes that are classified into two classes according to the structural features of their active site. The active site of Class I AaRS enzymes contain a catalytic Rossman fold with two conserved motifs, HIGH and KMSKS, whereas the active site of class II enzymes has an antiparallel β-sheet structure with three consensus motifs, I, II, and III in the catalytic center [14,15,16].

Methionyl tRNA synthetase (MetRS) is considered a class I AaRS enzyme expressing structural features of a class I aminoacyl-tRNA synthetase. Aside from the Rossmann fold and the signature sequences “HIGH” and “KMSKS” motifs, MetRS also contains connective peptide (CP) and a zinc finger along with the stem contact (SC) fold domain and C-terminal alpha-helix bundle domain [17,18].

Virtual screening (VS) is an effective low-cost CADD tool in drug discovery when compared with traditional high-throughput screening (HTS). Ligand-based and structure-based design are the two approaches for VS [19]. Molecular docking using crystal structures of target proteins from the protein data bank (PDB) is the most used method for structure-based drug design [19,20]. However, targets with unknown 3D crystal structure may require the use of a homology modeling strategy and pharmacophore modeling techniques. Structure-based or ligand-based pharmacophore could be used to obtain a 3D pharmacophore model [21]. As there is no crystal structure available for *P. mirabilis* MetRS, comparative structure modeling was used to construct a homology model for the *P. mirabilis* MetRS enzyme. The *P. mirabilis* MetRS homology model and its subsequent evaluation along with pharmacophore-based virtual screening is described.

## 2. Results and Discussion

### 2.1. Homology Model and Validation

Initial screening for possible templates for *P. mirabilis* MetRS amino acid sequence against the PDB-resolved structures was achieved using a BLAST analysis [22], obtained from the ExPASy proteomics server [23]. Three structures were identified and considered as possible templates (Table 1). For a structure to be considered a template, it should be a wild type, rather than engineered or mutant, have more than 25% of identity with the *P. mirabilis* MetRS amino acid sequence, and have the same function. The first three native hits were bacterial MetRS enzymes of *Escherichia coli* [24], that was the best template owing to the high sequence identity (80%), followed by *Acenitobacter baumannii* (59%), and *Pyrococcus abyss* (33%).

**Figure 2:**
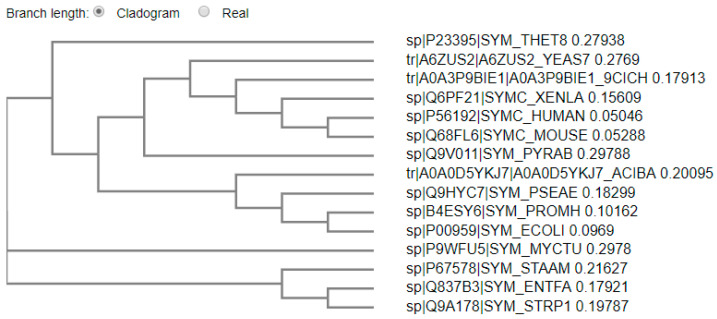
The phylogenetic tree of *Proteus mirabilus* MetRS (B4ESY6) in relation to other MetRS enzymes from: *Saccharomyces cerevisiae* (yeast) (A6ZUS2); human (P56192); mouse (Q68FL6); *zebra mbuna* (A0A3P9BIE1); African clawed frog (Q6PF21); *Mycobacterium tuberculosis* (P9WFU5); *Thermus thermophilus* (P23395); *Pseudomonas aeruginosa* (Q9HYC7); *Enterococcus faecalis* (Q837B3); *Streptococcus pyogenes* (Q9A178); *Escherichia coli* (P00959); *Acinetobacter baumannii* (A0A0D5YKJ7); *Pyrococcus abyssi* (Q9V011), *Staphylococcus aureus* (P67578).

To obtain more information regarding the best potential template, a phylogenetic tree was constructed using the phylogeny server [25] in order to determine the relative distances between various templates and the target sequence (Figure 2). The different evolutionary branching between the prokaryotic and eukaryotic MetRS enzymes is obviously demonstrated in the constructed phylogenetic tree. The closest homologies to *P. mirabilis* in this group of species was the Gram-negative bacteria *E. coli* (P00959) followed by *Pseudomonas aeruginosa* (Q9HYC7). A lower homology was observed with the Gram-positive *Enterococcus faecalis* and *Streptococcus pyogenes*, with a clear difference observed for the non-bacterial organisms. A percent identity matrix (Supplementary Figure S1) provided further validation.

### 2.2. Multiple Sequence and Structural Alignments

**Figure 3:**
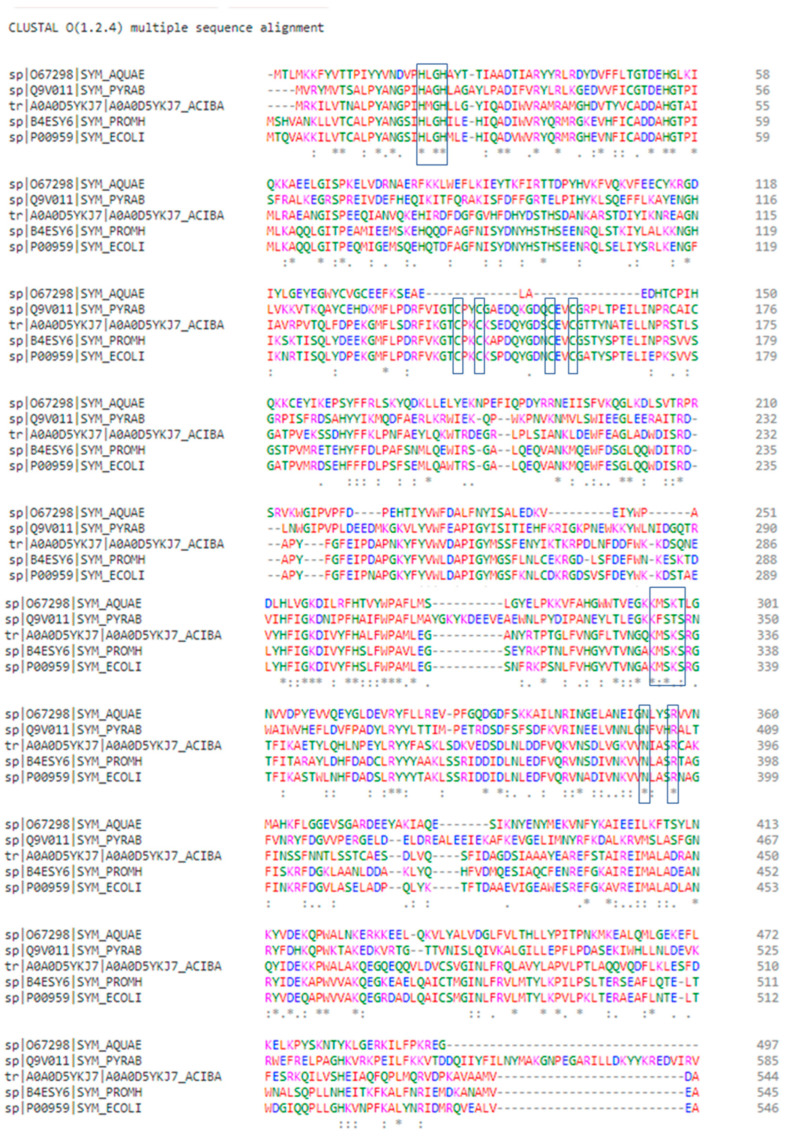

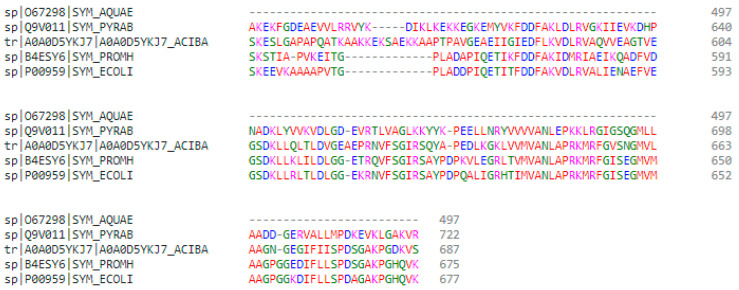
Sequence alignment of MetRS enzymes of *Aquifex aeolicus*, *Pyrococcus abyssi, Acinetobacter baumannii, Proteus mirabilus* and *Escherichia coli* using Clustal O in which “*” means that the residues are identical, “:” means that conserved substitutions have been observed, “.” means that semi-conserved substitutions are observed. The residues are colored according to their chemical properties where red, small hydrophobic (AVFPMILWY); blue, acidic (DE); purple, basic (RHK); green, hydroxyl + amine + basic (STYHCNGQ).

Clustal Omega 1.2.4 [26] was used to align the possible template sequences: *E. coli* (P00959), *A. baumannii* (A0A0D5YKJ7), *P. abyssi* (Q9V011), and *A. aeolicus* (O67298) MetRSs with the amino acid sequence of *P. mirabilis MetRS* (Figure 3). The input set of query and template sequences have an evolutionary relationship as they are Gram-negative bacteria. No gap was observed in alignment between the *P. mirabilis* and *E. coli* MetRS sequence and there were very few gaps in the sequence alignment of *P. mirabilis*, *P. abyssi* and *A. baumannii* MetRS. This result is consistent with the distinction between the two groups that is explained by the constructed phylogenetic tree. HIGH and KMSKS motifs are recognized in the proposed sequences of the possible templates and the query enzyme, indicated by the boxed amino acid residues in Figure 3.

The secondary structure prediction for *P. mirabilis* MetRS using PSIPRED [27] revealed the high helix content predicted throughout the sequence. The C-terminus had a higher degree of strands and coils as expected compared with the rest of the protein sequence. The region of the HIGH motif (Table 2) is a sequence of about ten residues and present in most homologous enzymes at the sequence positions just before the 22nd residue in the N terminus of these enzymes. This area of the query sequence was predicted by PSIPRED to fold in coils and helices (Supplementary Figures S2 and S3).

The KMSKS motif contains the ATP binding site and has been found in the *P. mirabilis* sequence aligned to the *E. coli* sequence (Table 2). The *E. coli* 3D structure contains coils and strands from position 333 to 337 which agrees with the PSIPRED predictions for the query sequence. The Rossmann fold domain of *E. coli*, formed by two polypeptide sequences (residues 6–115 and 252–326), is connected by the connective polypeptide domain (residues 119–251).

### 2.3. 3D Homology Model

**Figure 4:**
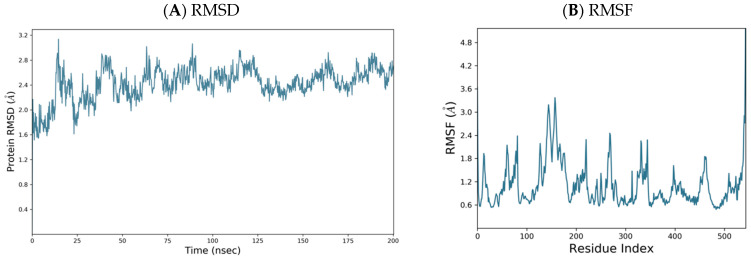
(**A**) RMSD and (**B**) RMSF plots for *P. mirabilis* MetRS over 200 ns molecular dynamics simulation.

The lowest energy 3D homology model was constructed using *E. coli* MetRS (1F4L) crystal structure through the Molecular Operating Environment (MOE) software [28], as explained in the experimental section. The homology model was subject to a 200 ns molecular dynamic (MD) simulation using the Desmond programme of Maestro (Schrödinger) [29,30]. The *P. mirabilis* MetRS homology model was equilibrated with a small change in Root Mean Square Deviation (RMSD) from 1.72 Å at 0 ns to 2.65 Å at 200 ns (Figure 4). Root Mean Square Fluctuation (RMSF) showed areas of higher fluctuation (the loop regions and N- and C-terminals) and areas of less fluctuation related to the secondary structural elements (SSE) such as α-helices and β-sheets (Figure 4), consistent with the 48.44% SSE (% helix 35.79, % strand 12.65) in *P. mirabilis* MetRS.

**Figure 5:**
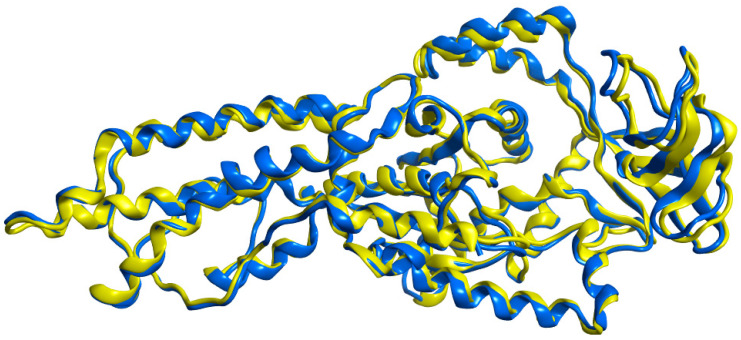
Superimposition of *E. coli* MetRS (1F4L) in yellow color with *P. mirabilis* MetRS model in blue color.

Superimposition of the *P. mirabilis* MetRS model with the main template, *E. coli* MetRS (1F4L), using MOE showed a low RMSD of 0.877 Å over 544 amino acid residues indicating a high degree of similarity (Figure 5).

### 2.4. Model Evaluation/Validation

**Figure 6:**
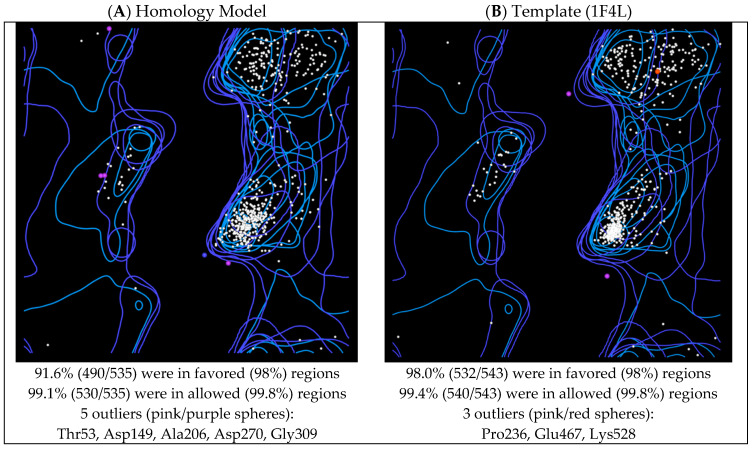
Ramachandran plots of (**A**) *P. mirabilis* MetRS homology model and (**B**) *E. coli* MetRS template (1F4L).

Ramachandran analysis using MolProbity [31] of the *P. mirabilis* MetRS homology model and the *E. coli* MetRS template (pdb 1F4L [32]) indicated that 99.1% (530/535) of amino acid residues of the homology model were in the allowed regions compared with 99.4% (540/543) in the template with five and three amino acids identified as outliers, respectively (Figure 6; Supplementary Figures S4 and S5).

**Figure 7:**
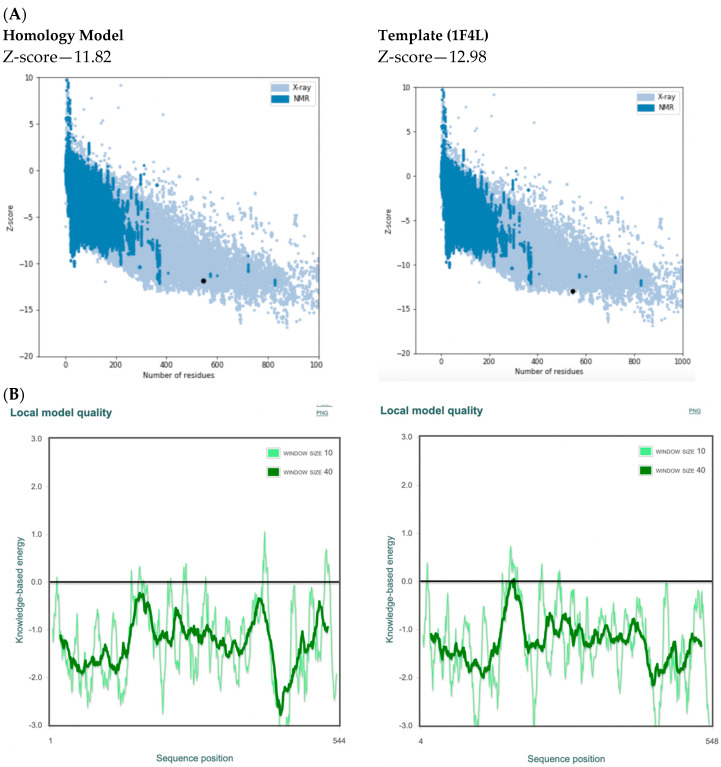
ProSA output showing: (**A**) overall model quality (z-score) of the *P. mirabilis* MetRS homology model and template *E. coli* MetRS (pdb 1F4L) in a plot that contains the z-scores of all experimentally determined protein chains in current PDB from different sources (X-ray, NMR) as distinguished by different colors and (**B**) local model quality plot.

The homology model performed well compared with the template 1F4L in the ProSA (protein structure analysis) evaluation [33] with a z-score of −11.82 compared with the template which had a z-score of −12.98 (Figure 7A). The local model quality plot of the homology morel shows no positive values, which would correspond with problematic or erroneous parts of the input structure, suggesting a good quality model (Figure 7B).

### 2.5. Active Site Validation and Docking

The predicted active site was validated by the Clustal O (1.2.4) multiple sequence alignment [26] and the alignment service from MOE [28] and by the docking of suitable ligands into the putative *P. mirabilis* MetRS model.

**Figure 8:**
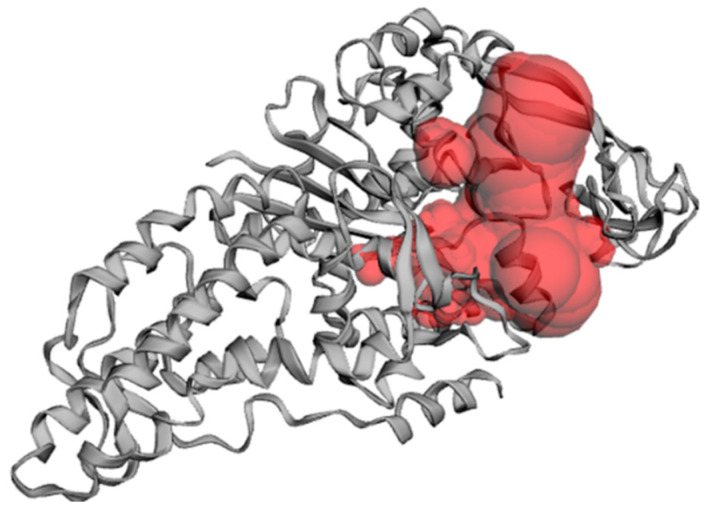
Cartoon representation of the binding site topology of the homology model. Putative pocket; red color, was calculated via the CASTP server of proteins.

Moreover, the binding pocket of the modelled protein structure was predicted by the Computed Atlas of Surface Topography of Proteins (CASTP) server [34]. Figure 8 shows the putative pocket of the homology model. The calculated Richards’ solvent accessible surface area and volume were estimated as 1833.297 Å^2^/3252.450 Å^3^, for the binding site.

The ligands include methionine, the natural substrate of the MetRS and, as *E. coli* MetRS is the closest homologue to *P. mirabilis* MetRS, the *E. coli* MetRS inhibitors trifluoromethionine (MF3), difluoromethionine (2FM), (1-amino-3-methylsulfanylpropyl)-phosphonic acid (MPH), methionine phosphinate (MPJ), 5′-*O*-[*N*-(L-methionyl)-sulfamoyl] (MSP), and methioninyl adenylate (MOD) were also used for the docking validation and analysis.

Protein–ligand complexes were generated from docking of these seven ligands (Table 3), in the *P. mirabilis* MetRS homology model, with the active site defined as selected amino acids (Ala12, Leu13, Pro14, Tyr15, Gly23, His24, Glu27, Asp52, Trp253, Ala256, Pro257, Tyr260, His300, His322, Tyr324, and Val325) identified as the methionyl-AMP pocket by alignment of the co-crystallized structure of *E. coli* MetRS with methionyl adenylate (pdb 1PG0) [32].

The protein–ligand complexes were then subject to 200 ns MD simulations using the Desmond programme of Maestro (Schrödinger) [29,30].

The smaller methionine and methionine derivatives (MPH, MPJ, 2FM, and MF3) showed fluctuation and changes in ligand RMSD (Supplementary Figure S6), possibly owing to the greater conformational flexibility of these small ligands. The most stable protein–ligand complex of the larger ligands, MSP and methionyl adenylate, was with the methionyl adenylate ligand while greater fluctuation and change in ligand RMSD was observed for MSP (Supplementary Figure S6).

**Figure 9:**
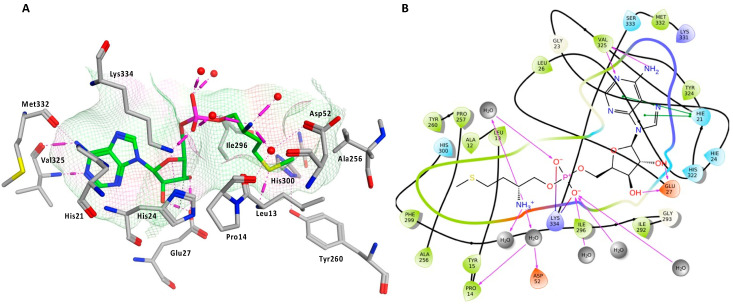
(**A**) 3D and (**B**) 2D images of *P. mirabilis* MetRS—methionyl adenylate (green) protein–ligand complex after MD simulation illustrating binding site and binding interactions.

In the methionyl adenylate protein–ligand complex, the methionine moiety sits in a pocket lined by Ala12, Leu13, Pro14, Tyr15, Asp52, Ala256, Pro257, Tyr260, Phe299, and His300, with binding observed between the thiol group and the backbone of Leu13 and direct and water-mediated H-bonding observed between the amine group with Asp52 and Pro14. A salt-bridge forms between Lys334 and the phosphate moiety, which also binds through water molecules and intramolecularly with the ligand amine group. The 2′- and 3′-hydroxy groups of the ribose ring form H-bonding interactions with Glu27, while the adenine forms H-bonding interactions through one N in the pyrimidine ring and the exocyclic amine with Val325 and face-edge π-π interaction with His21 (Figure 9).

**Figure 10:**
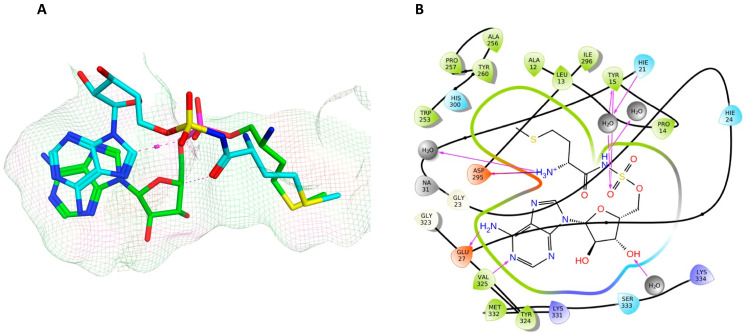
(**A**) Positioning of MSP (cyan) relative to methionyl adenylate (green) in *P. mirabilis* MetRS. (**B**) Two dimensional image of *P. mirabilis* MetRS—MSP protein–ligand complex after MD simulation illustrating binding interactions.

The MSP ligand showed a good overlap with methionyl adenylate in the methionine and phosphate regions (Figure 10); however, the ribose ring showed a significant change in conformation, which reflects the change in RMSD observed over the MD simulation (Table 3; Supplementary Figure S6), and loss of binding between Glu27 and the ribose hydroxy groups (Figure 10).

**Figure 11:**
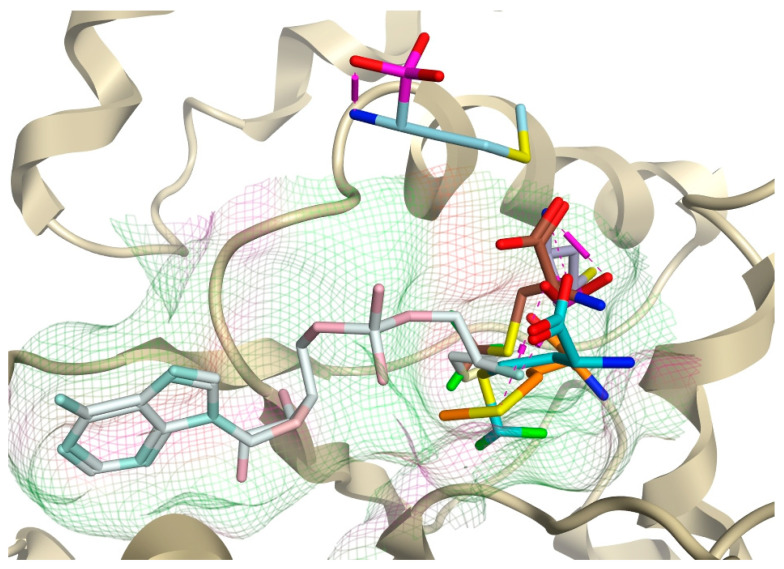
Positioning of methionine (orange), MPH (light blue), MPJ (light purple), 2FM (turquoise), and MF3 (brown) within the methionine binding site compared with adenylate methionine (white), which sits in the ATP and methionyl amino acid pockets.

The methionine–*P. mirabilis* MetRS ligand complex showed comparable placement and binding of methionine as observed for the methionyl moiety of methionyl adenylate (Figure 4), and this was also observed for MPJ, 2FM, MF3, and for MPH; however, MPH was observed in the same binding site only at 50 ns while at 100, 150, and 200 ns MPH was displaced outside the active site (Figure 11 and Figure S7) with the amine group of the ligand binding with Asp295 rather than Asp52 observed for methionine and the other methionine derivatives (Supplementary Figure S8). This MD study provides support for the validity of the AMP/methionine active sites. The model of *P. mirabilis*–MetAMP protein–ligand complex is available in ModelArchive at https://modelarchive.org/doi/10.5452/ma-h0jz3.

### 2.6. Recognition and Binding of P. mirabilis MetRS with Cognate tRNA^met^

In each living cell, the synthesis of protein usually starts with methionine, which is supplied to the ribosome as methionyl-tRNA^Met^ produced by MetRS. As a result, there are two types of the tRNA^Met^ (the initiator tRNA_f_^Met^ and the elongator tRNA_m_^Met^) and the MetRS acylates both, even though they have extremely diverse nucleotide sequences. Extensive biochemical studies [35] have shown cytosine at the anticodon position (C34) to primarily control the identity of tRNA^Met^ for L-methionine and the other two anticodon bases (A35 and U36) are the second most important identity factors [36].

Despite the simple architecture of MetRS, the crystal structure of tRNA^Met^ binding with *E. coli* MetRS has remained unresolved. The crystal structure of *Aquifex aeolicus* MetRS complexed with tRNA^Met^ has been reported [37] with Asn353, Arg357, and Trp422 of *Aquifex aeolicus* MetRS observed to be directly involved in the base specific recognition of the tRNA^Met^ anticodon. These three amino acids are strictly conserved in the MetRSs from eubacteria, archaea, and eukaryotes [37].

Proposed interactions of the CAU anticodon in tRNA^Met^ with *P. mrabilis* MetRS residues are based on the Clustal alignment of the query enzyme with *E. coli* and *A. aeolicus* (Figure 3) where boxed amino acids residues, present at the C-terminus, are responsible for recognition and interaction with tRNA^Met^ anticodon bases (Table 4).

**Figure 12:**
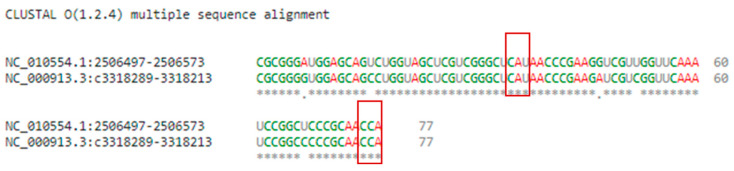
Genomic alignment of tRNA^met^ for *P. mirabilis* and *E. coli*, respectively, where boxed nucleotides are the conserved CAU anticodon and CCA end. “*” indicates identical nucleotide.

The *P. mirabilis* tRNA^Met^ sequence, available from the National Centre for Biotechnology Information consists of 77 bases, comparable with *E. coli*. Moreover, the CAU anticodon positions are also the same (Figure 12).

**Figure 13:**
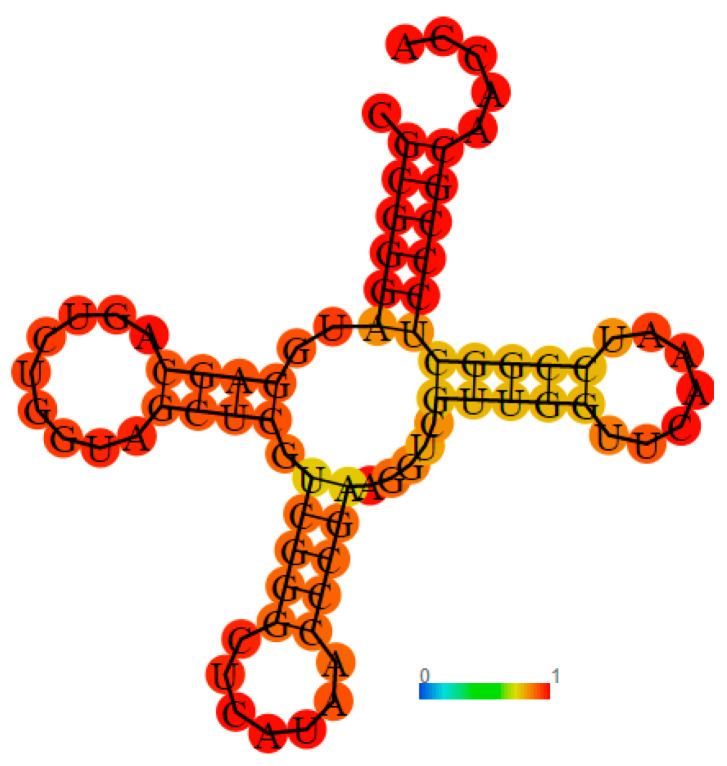
Cloverleaf representation of predicted secondary structure of the *P. mirabilis* tRNA^Met^.

Using the RNA fold server [38], the tRNA^Met^ secondary structure of *P. mirabilis* was predicted (Figure 13).

Asn390, Arg394, and Trp460 of *P. mirabilis* MetRS are involved in binding the anticodon part of Met-tRNA. The corresponding amino acid residues in the MetRSs of *A. aeolicus* and *E. coli* play the same role. These outcomes are predictable from the literature and sequence alignments.

### 2.7. Systematic Dataset Preparation

**Figure 14:**
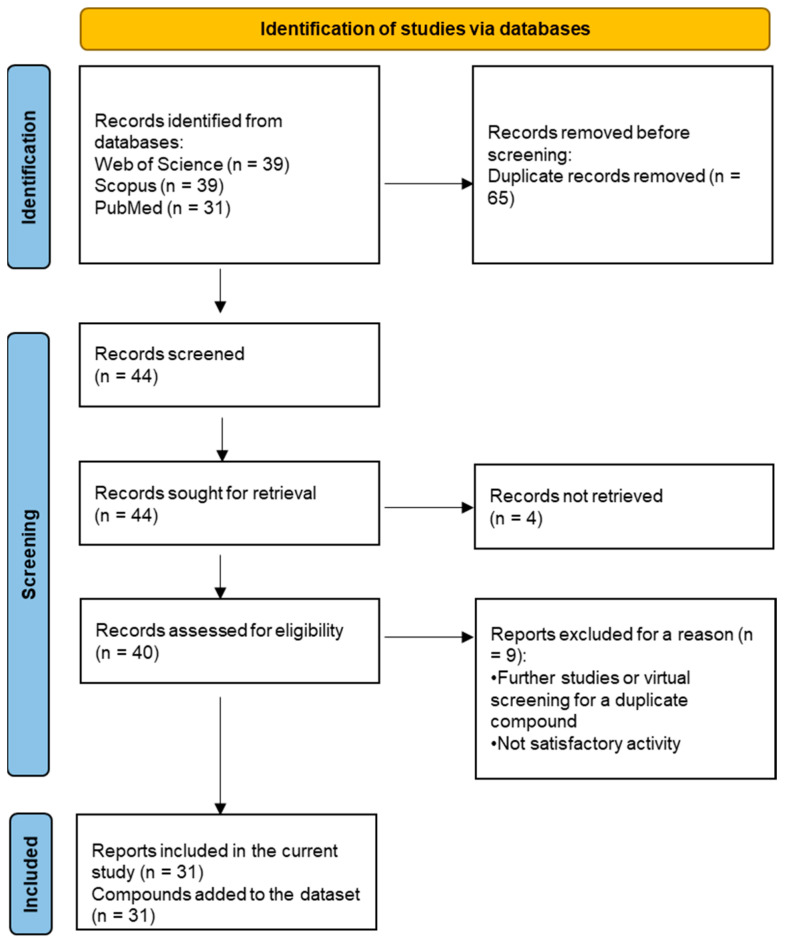
PRISMA 2020 flow diagram for compound selection process through database searches.

A systematic database search was used to select active compounds against MetRS in a variety of organisms to prepare a dataset of compounds for *P. mirabilis* MetRS screening. The selection process followed the PRISMA flowchart [39], as summarized in Figure 14.

The database search resulted in a total of 109 publications. Elimination of duplicates resulted in 44 publications with MetRS inhibitory activity against different organisms. These publications were retrieved and studied to ensure elimination of duplicate and nonactive compounds. The selection process of one or two of the most active compounds in each relevant publication resulted in a dataset of thirty-one compounds summarized in Table 5.

### 2.8. Pharmacophore-Based Virtual Screening

**Figure 15:**
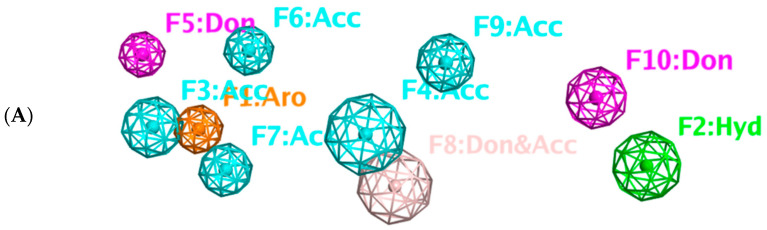

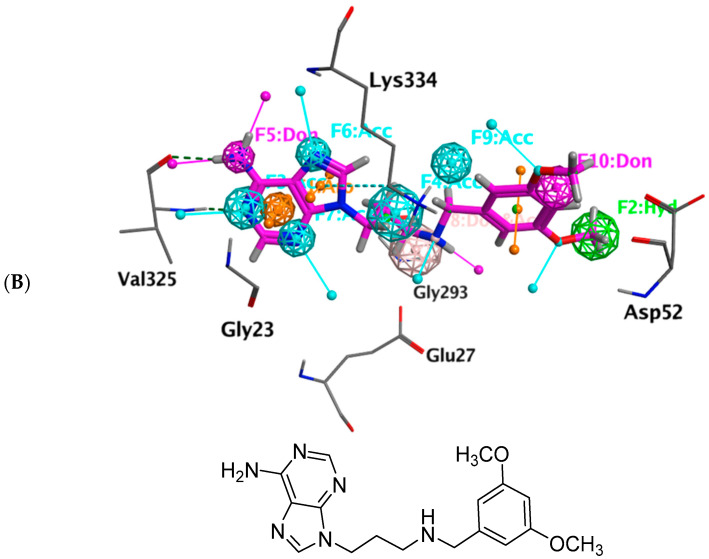
Pharmacophore-based virtual screening: (**A**) 3D pharmacophore with ten features; (**B**) the most promising compound fitting with the pharmacophoric features in the active site of the model.

Three compounds of the prepared library are inhibitors for *E. coli* MetRS [54,55,56,63,64]. These compounds along with the natural substrate were used to prepare a 3D pharmacophore model within MOE [28]. A ten-feature model was generated (Figure 15A) and used to screen the created library. Initial screening with minimum three features resulted in 29 of the 31 compounds passing the pharmacophoric filter. Further analysis with minimum four features matched, and a visual inspection of the binding resulted in the choice of one compound as the most promising inhibitor (Figure 15B) [67]. This compound was able to match most of the features represented by having an aromatic center (F1), hydrophobic centroid (F2), several hydrogen bond donor and acceptor features (F3, F5, F6 and F7), and very close to the donor feature (F10). All these features made the compound able to fit perfectly in the pocket-forming interactions with some of the key amino acid residues (Val325, Lys334, and Leu13) to anchor the ligand.

Overall, the constructed model of *P. mirabilis* MetRS (Supplementary Figure S9) shows the characteristic domains of an AaRS class I enzyme. The model contained 544 amino acid residues from the 675 residues of the sequence, owing to the disabling of the C-terminus in the crystal structure of the main template. The secondary structure of these amino acid residues involved 24 α-helices and 21 β-sheets (Supplementary Figure S3). The architecture of the active site and the key amino acid residues Val325, Glu27, Lys334, Leu13, and Asp52 necessitate the presence of key pharmacophoric features as indicated in the aromatic center (F1), hydrophobic centroid (F2), and several possibilities for hydrogen bonding in the distance between them. Compounds that are able to fit and fill the space between F1 and F2 with hydrogen bond donors and acceptors in the proper orientation could have the possibility of inhibiting the enzymatic activity.

## 3. Materials and Methods

### 3.1. Homology Search

The *P. mirabilis* MetRS amino acid sequence was obtained from the ExPASy proteomics server at the Swiss Bioinformatics Institute [23]. The sequence of the enzyme has the Uniprot identifier B4ESY6 (SYM_PROMH) and is formed of 675 residues [71]. A homology search was performed using SIB BLAST service [22,72] accessible through the ExPASy server, which was used to align the *P. mirabilis* MetRS amino acid sequence against the sequences of the 3D-resolved structures in the protein data bank [73] to identify the best homologous proteins. The alignment parameters and the thresholds, which were used for screening expected homologues, were used with their default values and BLOSUM62 comparison matrix. The detailed parameters were described as the following: BLOSUM62 comparison matrix is utilized for protein with amino acids more than 85. E-expectation value threshold (E-value) is 10. The phylogeny server [25] was used to construct a phylogenetic tree for the target protein along with the possible predicted homologous proteins and other selected MetRS enzymes from diverse organisms.

### 3.2. Multiple Sequence and Structure Alignment

The query enzyme sequence was aligned with the protein sequences of the most related MetRS templates: *Escherichia coli* (pdb: 1F4L), *Acenitobacter baumannii* (pdb: 5URB), and *Pyrococcus abyssi* (pdb: 1RQG), using Clustal Omega 1.2.4 [26]. The local alignment of these sequences is useful to detect the conserved residues and the structural motifs: HIGH region, KMSKS motifs together with the zinc metal and the ATP binding sites. The revealed results are vital to understand not only the expected structural similarities but also the functional similarities between these enzymes. The secondary structure of *P. mirabilis* MetRS and the closest template (1F4L) were determined using PSIPRED v4.0 [27].

### 3.3. 3D Model Building

The molecular experiments were accomplished using Molecular Operating Environment (MOE) 2019.0102 molecular modeling software [28]. Homology models were constructed using MOE-Homology using AMBER99 forcefield [74], which uses a dictionary to set the partial charges of atoms in amino acids. The final homology model was constructed using the *E. coli* MetRS (1F4L) crystal structure. Ten intermediate models were generated, and the final model was taken as the Cartesian average of all the constructed intermediate models. All minimizations were performed until RMSD gradient of 0.05 kcal mol^−1^Å^−1^ with the specified forcefield and partial charges automatically calculated.

### 3.4. Model Validation

Stereochemical quality of the polypeptide backbone and side chains was assessed using the MolProbity server [31] via assessing both residue-by-residue geometry and overall structural geometry. The ProSA server [33] was used to check defaults in the three-dimensional structure of the protein based on statistical analysis. Validation data from the template (1F4L) were used as the baseline to evaluate the model.

### 3.5. Molecular Dynamics and Docking Studies

Methionine, as the natural substrate, was built as a ligand through MOE-Builder [28]. Structures of ligands were acquired from the relevant complex *E. coli* crystal structures (PDB codes: 1PFW (MF3), 1PFV (2FM), 1P7P (MPH), 1PFU (MPJ), 1PFY (MSP), and 1PG0 (MOD) and then each ligand’s energy was minimized, and a ligand database was generated.

Docking and molecular dynamics simulations were performed as previously described [75,76]. Docking studies, using the constructed model, were performed to generate protein–ligand complexes, using MOE [28] until a RMSD gradient of 0.01 kcal mol^−1^ Å^−1^ with the MMFF94 forcefield (ligands) and partial charges were automatically calculated. The active site was defined using the site finder in MOE. Additionally, the Computed Atlas for Surface Topography of Proteins (CASTP) server was utilized for prediction of the active pockets of the protein, using a 1.4 Å probe and at default settings [34]. Docking was performed using the Alpha Triangle placement to determine the poses, refinement of the results was performed using the MMFF94 forcefield, and rescoring of the refined results was completed using the London ΔG scoring function.

MD simulations were run on the protein–ligand complexes using the Desmond programme of Schrödinger [29,30]. Overlapping water molecules were deleted, and the systems were neutralized with Na^+^ ions and salt concentration of 0.15 M. Force-field parameters for the complexes were assigned using the OPLS_2005 forcefield, that is, a 200 ns molecular dynamic run in the NPT ensemble (T 1⁄4 300 K) at a constant pressure of 1 bar. Energy and trajectory atomic coordinate data were recorded at each 1.2 ns. The model of *P. mirabilis*–MetAMP protein–ligand complex is available in ModelArchive at https://modelarchive.org/doi/10.5452/ma-h0jz3.

Prime/MMGBAS, available in Schrödinger Prime suite, was used to calculate the binding free energy of the ligands with *P. mirabilis* MetRS model.

ΔG (bind) = E_complex (minimized) − (E_ligand (minimized) + E_receptor (minimized))

The mean ΔG (bind) values were calculated from each 10 frames of the final 100 ns of the 200 ns MD simulation (frames 500–1000), and the average generated ΔG was from each energy minimized frame using the equation shown above.

### 3.6. Systemic Dataset Preparation

The three databases, Web of Science, Scopus, and PubMed, were searched through the advanced search tool. Methionyl tRNA synthetase or MetRS, and inhibitors or derivatives were used as determinant keywords. To be eligible, the article had to be an original research article, available in the English language. The selected article must contain biological evaluation data against MetRS. The most active compound(s) was selected to be added to the database.

### 3.7. Pharmacophore Query

The system was prepared using the default settings for MOE [28], specifically the Amber10:EHT forcefield, solvation R-filed, and Receptor strength 5000. The docking poses for the ligands were loaded and superimposed with common features selected from the consensus to form the pharmacophore features (Figure 15A). Relevant features were selected. The search allowed for a partial match with a minimum of three or four features with best match determined from the rscore value (the sum of the individual feature (F1–F10) rscores) and visual inspection of fit and binding in the active site.

## 4. Conclusions

In silico development of a *P. mirabilis* MetRS homology model has been explored using *E. coli* MetRS as a template. The constructed model topology and domains were compatible with the characteristics of Class I AaRS enzymes. Docking and molecular dynamics simulations of the selected ligands provide the validity of the AMP/methionine active site. In the methionyl adenylate protein–ligand complex, the methionine moiety sits in a pocket lined by Ala12, Leu13, Pro14, Tyr15, Asp52, Ala256, Pro257, Tyr260, Phe299, and His300, with binding observed between the thiol group and backbone of Leu13 and direct and water mediated H-bonding observed between the amine group with Asp52 and Pro14. A salt-bridge forms between Lys334 and the phosphate moiety, which also binds through water molecules and intramolecularly with the ligand amine group. Asn390, Arg394, and Trp460, at the C-terminal, recognize and interact with tRNA^Met^ anticodon bases. Exploring the complete putative model with its active binding site and the key binding amino acid residues served as a crucial step in the development of novel antibiotics with greater selectivity using rational drug design. The prepared model was used to apply a virtual screening approach on a systematically prepared library of compounds and demonstrated applicability in finding promising compounds to be used as lead compounds for *P. mirabilis* MetRS inhibitors. The architecture of the active site and the main key amino acid residues Val325, Glu27, Lys334, Leu13, and Asp52 necessitated the presence of key pharmacophoric features as indicated in the aromatic center (F1), hydrophobic centroid (F2), and hydrogen bonding in the distance between these centers. The confirmation of the findings through laboratory experiments will be considered in a further study.

## Figures and Tables

**Table 1:** The first three hits in the *P. mirabilis* MetRS BLAST results.

Organism	PDB Code	BLAST ^a^ Score	Sequence Identity ^b^	Sequence Identity %	Positive %	Chain Length	E-Value
*E. coli*	1F4L	964	443/551	80	91	551	0.0
*A. baumannii*	5URB	697	319/544	59	77	567	0.0
*P. abyss*	1RQG	380	243/727	33	51	722	2 × 10^−120^

^a^ The score of BLAST for an alignment is calculated by summing the scores for each aligned position and the scores for gaps. ^b^ (Number of identical residues)/(length of sequence fragment identified by PSI-BLAST).

**Table 2:** Key amino acid residues of the main template (1F4L).

PDB	HIGH Region Motif	KMSKS Motif	Zinc Binding Residues	ATP Binding
1F4L	15–25	333–337	145, 148, 158 and 161	336

**Table 3:** Ligand structures and protein–ligand (P/L) RMSD at 0 and 200 ns.

Ligand	Structure	P/L RMSD (Å) 0 ns	P/L RMSD (Å) 200 ns
Methionine	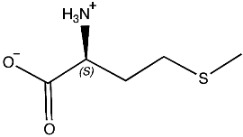	2.14/1.50	3.74/6.80
MPH	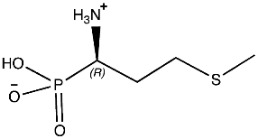	1.46/1.18	4.00/6.67
MPJ	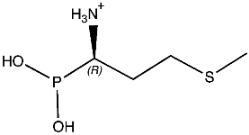	2.44/1.97	2.84/6.49
2FM	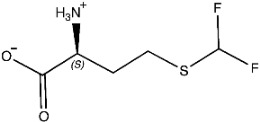	1.59/1.79	2.57/5.29
MF3	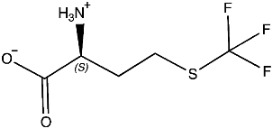	1.36/2.72	4.08/3.17
MSP	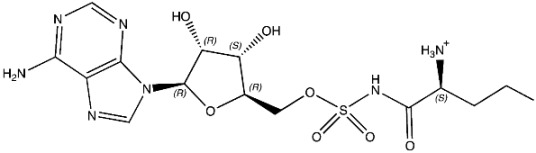	1.15/2.52	2.65/7.56
Methionyl adenylate	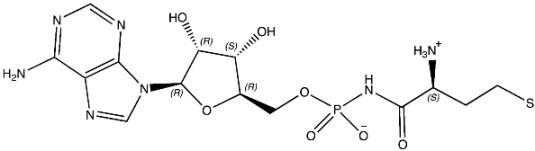	1.92/3.63	2.59/4.36

**Table 4:** Amino acids responsible for in the base specific recognition of the tRNA^Met^ anticodon.

*A. aeolicus*	*E. coli*	*P. mirabilis*
Asn356	Asn391	Asn390
Arg360	Arg395	Arg394
Trp430	Trp461	Trp460

**Table 5:** The dataset of compounds and their original target organism as reported.

Compound	Target Organism	Ref.
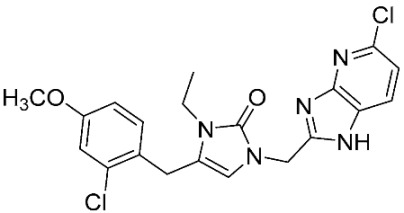	*Trypanosoma brucei*	[40]
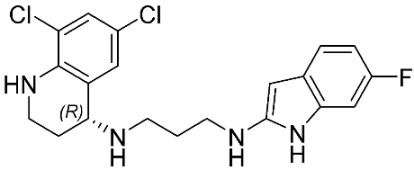	*Giardia lamblia*	[41,42]
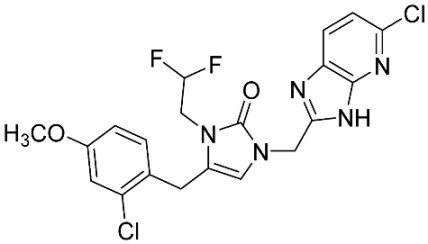	*Cryptosporidium*	[43]
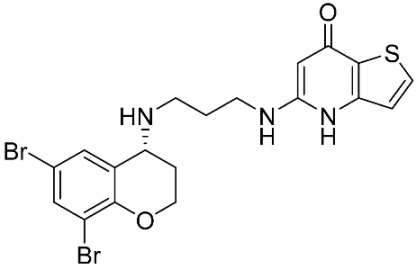	*Clostridium difficile*	[44]
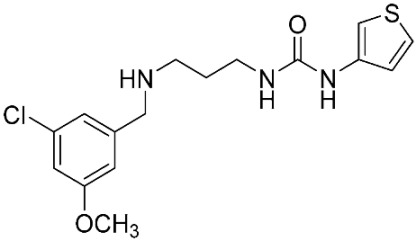	*Trypanosoma brucei*	[45,46]
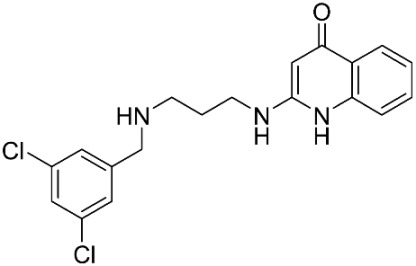	*Trypanosoma brucei*	[47,48]
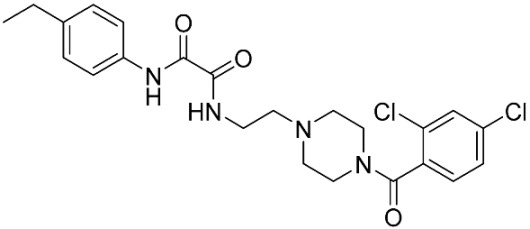	*MRSA/Staphylococcus aureus*	[49]
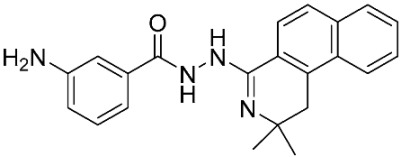	*Pseudomonas aeruginosa*	[49]
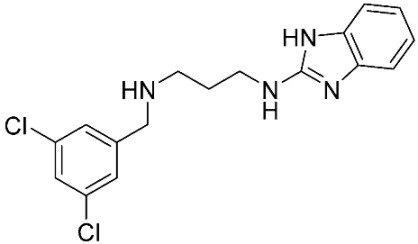	*Trypanosoma brucei*	[50]
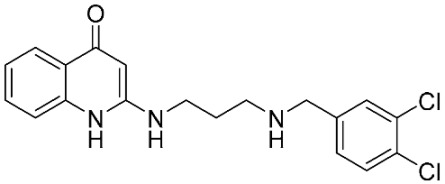	*Staphylococcus aureus*	[51]
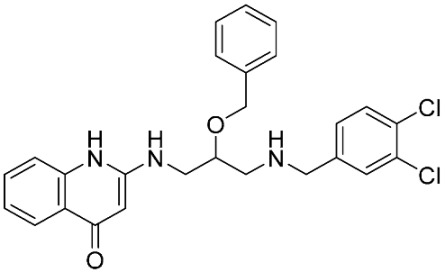	*Staphylococcus aureus*	[51]
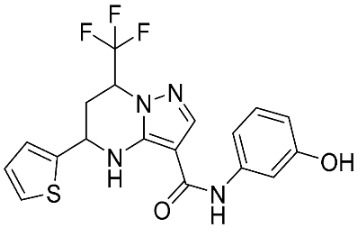	*Staphylococcus aureus*	[52]
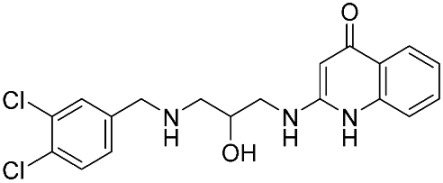	*Staphylococcus aureus*	[53]
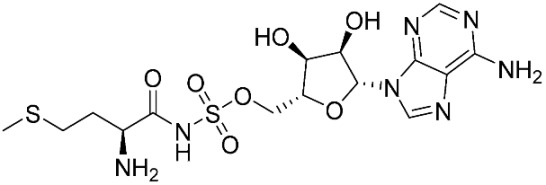	*Escherichia coli*	[54,55,56]
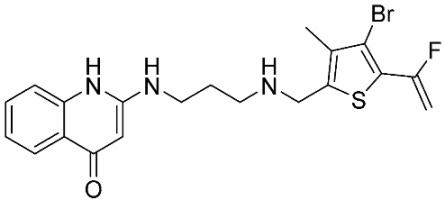	*Staphylococcus aureus*	[57]
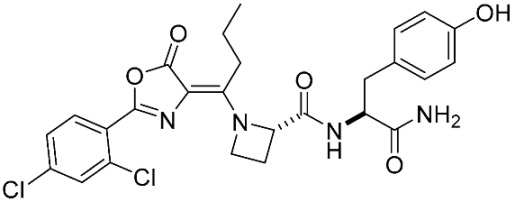	*Staphylococcus aureus*	[58]
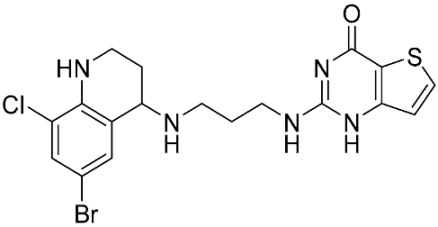	*Staphylococcus aureus*	[59]
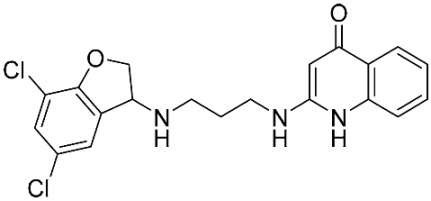	*Staphylococcus aureus*	[60]
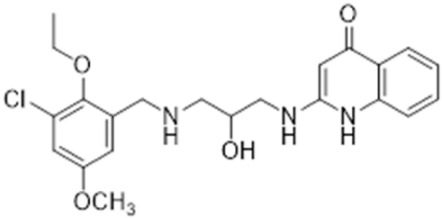	*Staphylococcus aureus*	[60]
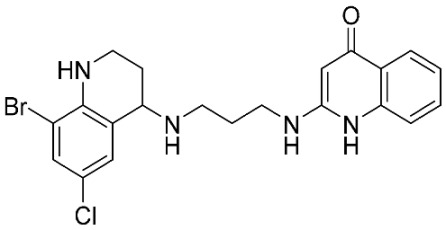	*Staphylococcus aureus*	[61]
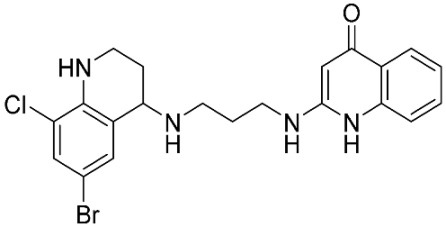	*Staphylococcus aureus*	[61]
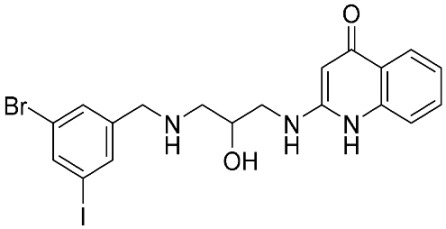	*Staphylococcus aureus*	[61]
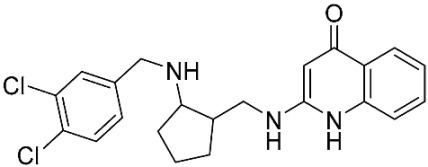	*Staphylococcus aureus*	[62]
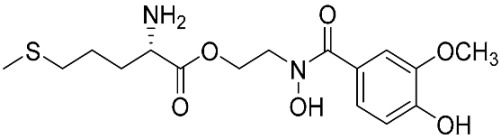	*Escherichia coli*	[63]
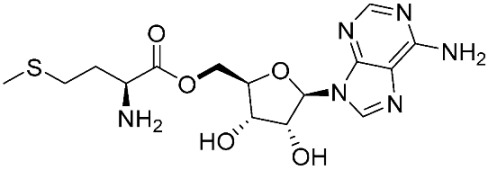	*Escherichia coli*	[64]
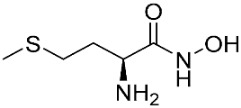	*Escherichia coli*	[65]
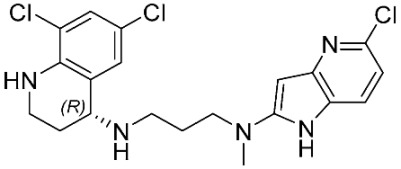	*Trypanosoma brucei*	[66]
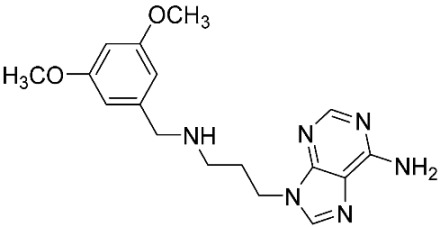	*Clostridium difficile*	[67]
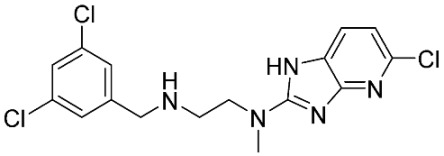	*Trypanosoma brucei*	[68]
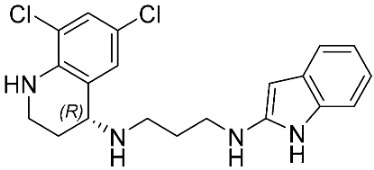	*Trypanosoma brucei*	[69]
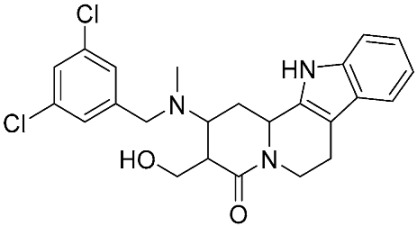	*Trypanosoma brucei*	[70]

## Data Availability

Data are contained within this article and in the Supplementary Materials.

## Supplementary Materials

The following supporting information can be downloaded at: https://www.mdpi.com/article/10.3390/ph16091263/s1, Figure S1: percent identity matrix created by Clustal alignment with *P. mirabilis* MetRS highlighted; Figure S2: amino acid sequence of *P. mirabilis* MetRS presenting α-helices in pink color, β-sheets in yellow color, loops in grey color; Figure S3: predicted query *P. mirabilis* MetRS sequence secondary structure; Figure S4. Ramachandran plots—*P. mirabilis* MetRS homology model (MolProbability); Figure S5. Ramachandran plots—*E. coli* MetRS template (1F4L) (MolProbability); Figure S6. RMSD plots of protein–ligand complexes over 200 ns MD simulation; Figure S7. 2D ligand interactions of the final frame of the protein–ligand complexes after 200 ns MD simulation; Figure S8. (A) change in position of MPH over 200 ns MD simulation: 50 ns (light pink), 100 ns (green), 150 ns (orange) and 200 ns (turquoise). (B) 2D ligand interactions of the *P. mirabilis* MetRS-MPH complex at 50, 100, 150 and 200 ns MD simulation; Figure S9: final *P. mirabilis* MetRS homology model with the characteristic domains in ribbon representation: Rossmann fold: red; connective peptide (CP): green; KMSKS domain: yellow; anticodon domain: purple.
