# Supplementary material for: Exploring Proteus mirabilis Methionine tRNA Synthetase Active Site: Homology Model Construction, Molecular Dynamics, Pharmacophore and Docking Validation
Source: Pharmaceuticals (Basel). 2023 Sep 6;16(9):1263. doi: 10.3390/ph16091263 (PMC10535265; doi:10.3390/ph16091263)
Supplement: Supplementary file 1 [file pharmaceuticals-16-01263-s001.zip › pharmaceuticals-2536779-supplementary.pdf]

Supplementary Data

Exploring *Proteus mirabilis* MetRS active site: homology model construction, molecular dynamics, pharmacophore and docking validation

Samar S. Elbaramawi [1], Ahmed G. Eissa[1], Nada A. Noureldin [1] and Claire Simons [2, *]

1 Department of Medicinal Chemistry, Faculty of Pharmacy, Zagazig University, Zagazig, Egypt, P.C. 44519; sselbaramawy@pharmacy.zu.edu.eg (S.S.E.); ageissa@pharmacy.zu.edu.eg (A.G.E.); nanoureddine@pharmacy.zu.edu.eg (N.A.N.)

2 School of Pharmacy & Pharmaceutical Sciences, Cardiff University, King Edward VII Avenue, Cardiff CF103NB, UK; simonsc@cardiff.ac.uk

* Correspondence: C. S.: simonsc@cardiff.ac.uk

Table of content:

Content	Page No.
**Figure S1.** Percent identity matrix created by Clustal alignment with *P. mirabilis* MetRS highlighted (red box)	S2
**Figure S2**. Amino acid sequence of *P. mirabilis* MetRS presenting α-helices in pink colour, β-sheets in yellow colour, loops in grey colour.	S3
**Figure S3.** Predicted query *P. mirabilis* MetRS sequence secondary structure.	S4
**Figure S4**. Ramachandran plots – *P. mirabilis* MetRS homology model (MolProbability)	S5
**Figure S5**. Ramachandran plots – *E. coli* MetRS template (1F4L) (MolProbability)	S6
**Figure S6**. RMSD plots of protein-ligand complexes over 200 ns MD simulation.	S7
**Figure S7**. 2D ligand interactions of the final frame of the protein-ligand complexes after 200 ns MD simulation.	S8
**Figure S8**. (A) Change in position of MPH over 200 ns MD simulation: 50 ns (light pink), 100 ns (green), 150 ns (orange) and 200 ns (turquoise). (B) 2D ligand interactions of the *P.mirabilis* MetRS-MPH complex at 50, 100, 150 and 200 ns MD simulation.	S9
**Figure S9:** Final *P. mirabilis* MetRS homology model with the characteristic domains in ribbon representation: Rossmann fold: red, connective peptide (CP): green, KMSKS domain: yellow and anticodon domain: purple.	S10

1: sp|P23395|SYM_THET8	100.00	42.02	41.24	42.48	39.84	23.12	24.19	24.91	24.24	24.06	31.67	28.14	25.80	27.01	26.85
2: sp|P9WFU5|SYM_MYCTU	42.02	100.00	40.70	41.44	40.74	20.51	23.73	23.47	24.02	24.02	27.13	22.85	24.14	23.99	24.95
3: sp|P67578|SYM_STAAM	41.24	40.70	100.00	58.87	55.38	21.10	22.69	24.45	23.82	23.82	27.74	25.08	24.96	28.04	28.32
4: sp|Q837B3|SYM_ENTFA	42.48	41.44	58.87	100.00	62.29	23.43	23.42	24.50	24.04	25.04	28.24	26.97	26.50	27.96	28.39
5: sp|Q9A178|SYM_STRP1	39.84	40.74	55.38	62.29	100.00	23.66	23.24	24.50	25.04	24.37	27.13	26.27	25.40	27.50	27.78
6: tr|A6ZUS2|A6ZUS2_YEAS7	23.12	20.51	21.10	23.43	23.66	100.00	46.20	45.17	44.00	43.47	35.90	28.81	29.83	28.14	27.77
7: tr|A0A3P9BIE1|A0A3P9BIE1_9CICH	24.19	23.73	22.69	23.42	23.24	46.20	100.00	64.86	63.90	63.41	34.41	27.56	28.50	27.27	27.89
8: sp|Q6PF21|SYMC_XENLA	24.91	23.47	24.45	24.50	24.50	45.17	64.86	100.00	68.27	68.34	34.72	28.41	27.05	26.97	27.42
9: sp|P56192|SYMC_HUMAN	24.24	24.02	23.82	24.04	25.04	44.00	63.90	68.27	100.00	89.67	34.30	27.08	27.03	27.91	27.24
10: sp|Q68FL6|SYMC_MOUSE	24.06	24.02	23.82	25.04	24.37	43.47	63.41	68.34	89.67	100.00	34.79	27.24	26.20	26.74	26.25
11: sp|Q9V011|SYM_PYRAB	31.67	27.13	27.74	28.24	27.13	35.90	34.41	34.72	34.30	34.79	100.00	33.09	32.14	34.68	34.43
12: tr|A0A0D5YKJ7|A0A0D5YKJ7_ACIBA	28.14	22.85	25.08	26.97	26.27	28.81	27.56	28.41	27.08	27.24	33.09	100.00	61.90	57.70	58.72
13: sp|Q9HYC7|SYM_PSEAE	25.80	24.14	24.96	26.50	25.40	29.83	28.50	27.05	27.03	26.20	32.14	61.90	100.00	62.46	65.48
14: sp|B4ESY6|SYM_PROMH	27.01	23.99	28.04	27.96	27.50	28.14	27.27	26.97	27.91	26.74	34.68	57.70	62.46	100.00	80.15
15: sp|P00959|SYM_ECOLI	26.85	24.95	28.32	28.39	27.78	27.77	27.89	27.42	27.24	26.25	34.43	58.72	65.48	80.15	100.00

Figure S1.

Percent identity matrix created by Clustal alignment with *P. mirabilis* MetRS highlighted (red box)

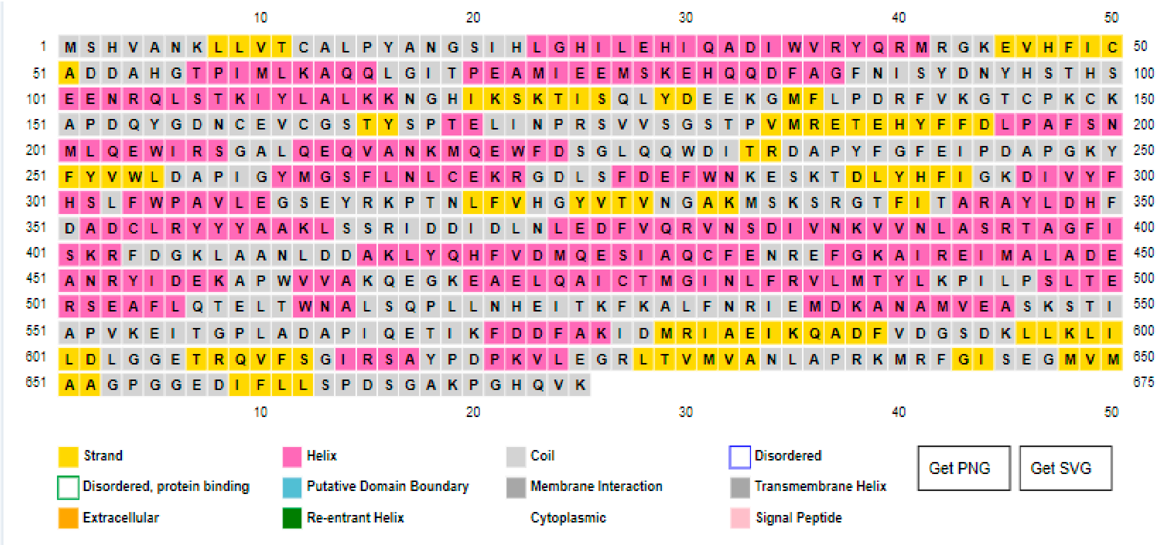

Figure S2. Amino acid sequence of *P. mirabilis* MetRS presenting α-helices in pink colour, β-sheets in yellow colour, loops in grey colour.

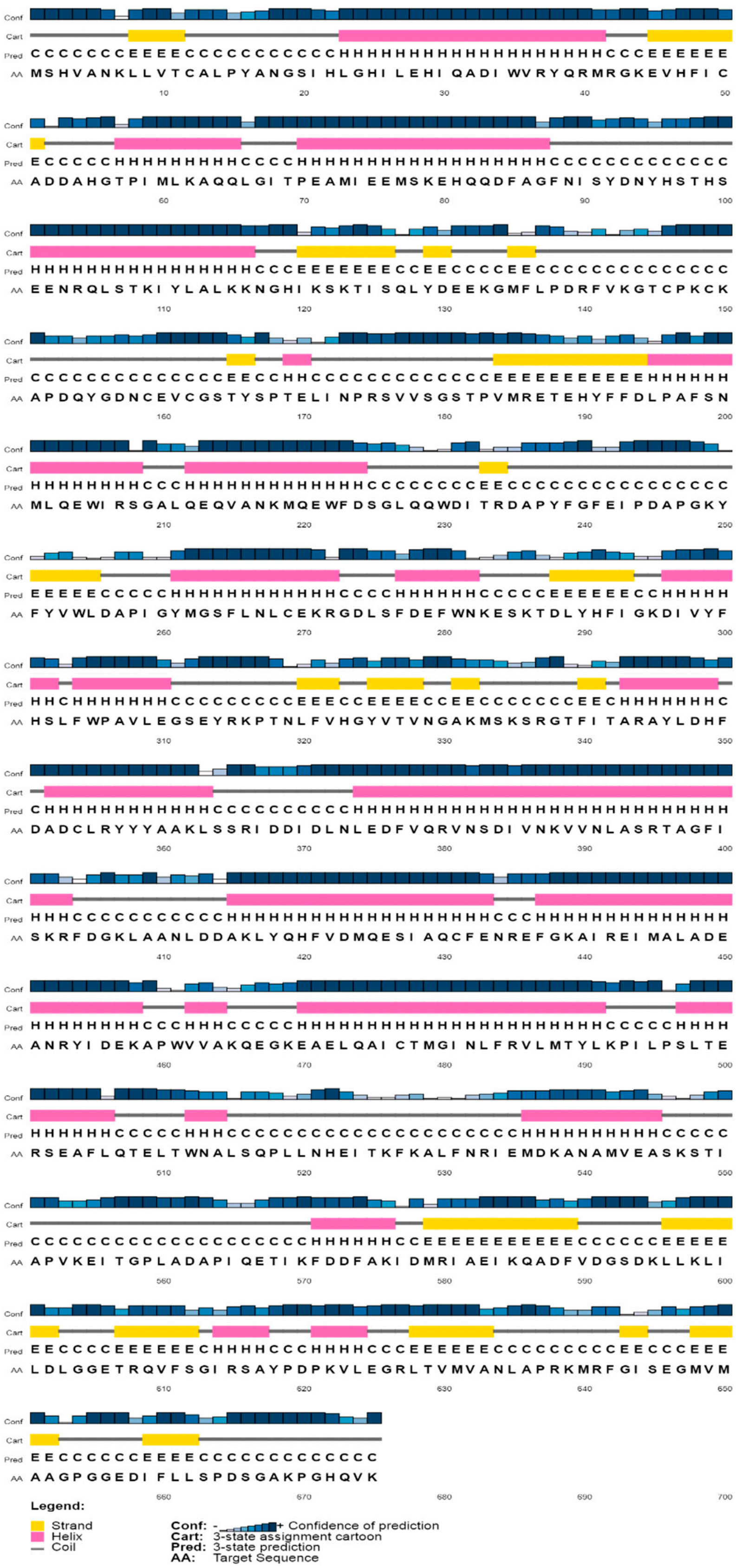

Figure S3. Predicted query *P. mirabilis* MetRS sequence secondary structure.

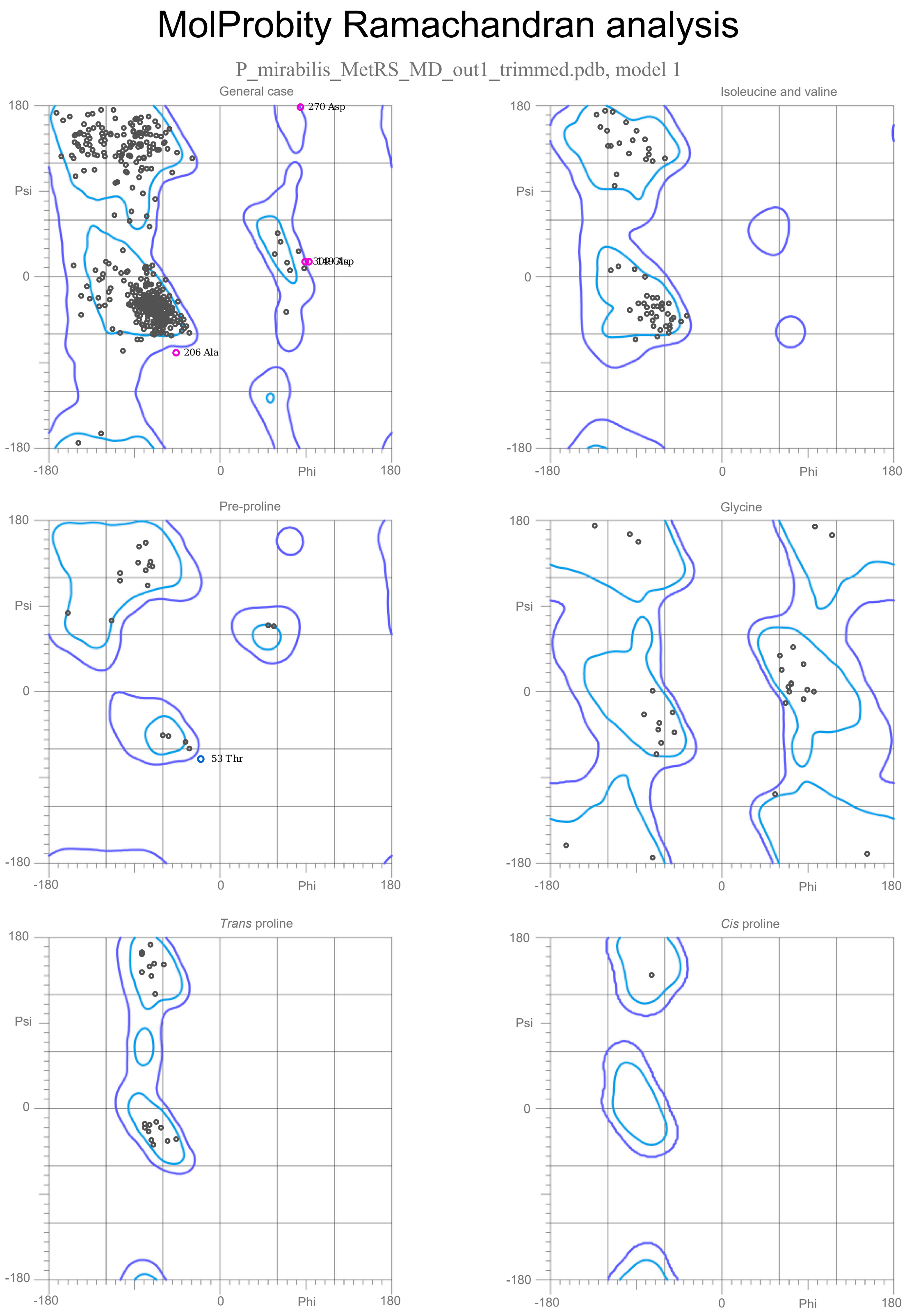

Figure S4. Ramachandran plots – *P. mirabilis* MetRS homology model (MolProbability)

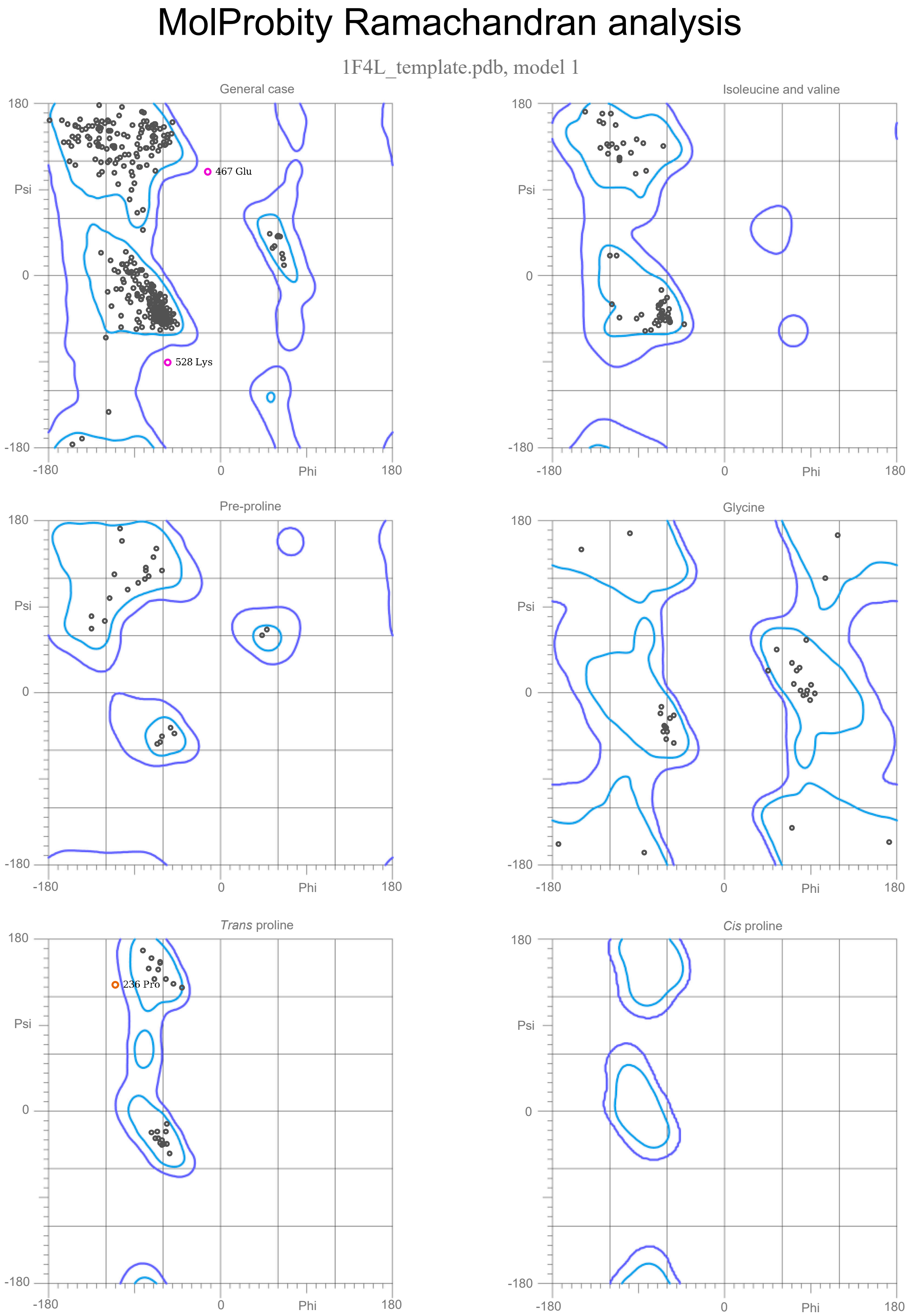

Figure S5. Ramachandran plots – *E. coli* MetRS template (1F4L) (MolProbability)

Methionine

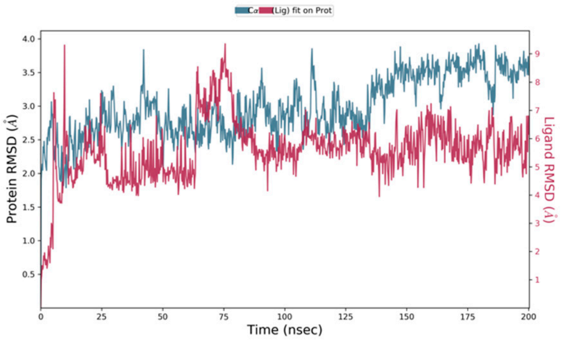

MPH

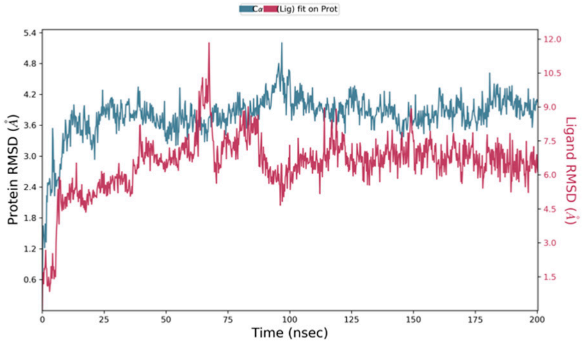

MPJ

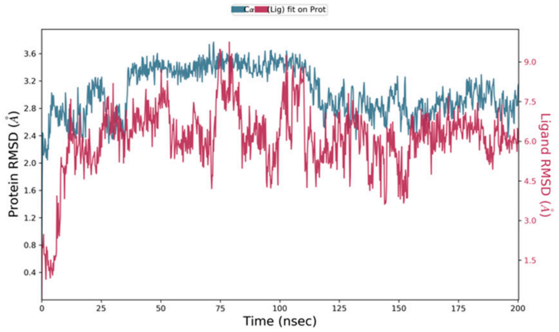

2FM

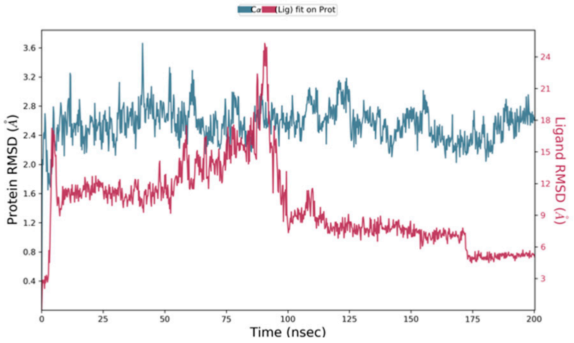

MF3

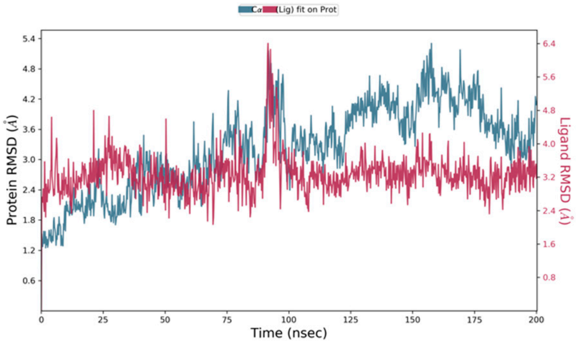

MSP

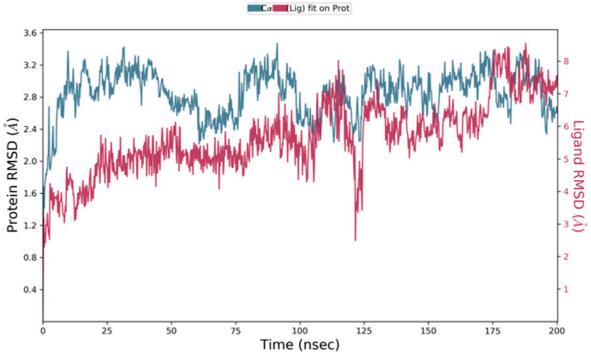

Methionyl adenylate

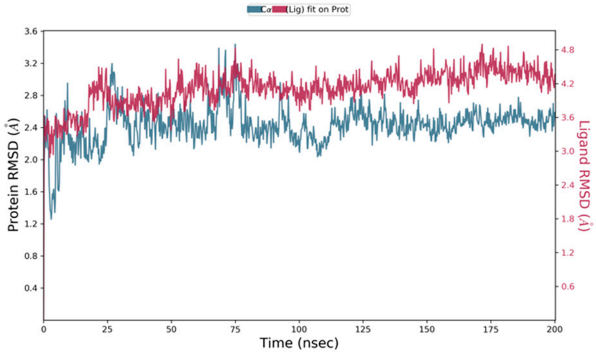

Figure S6. RMSD plots of protein-ligand complexes over 200 ns MD simulation.

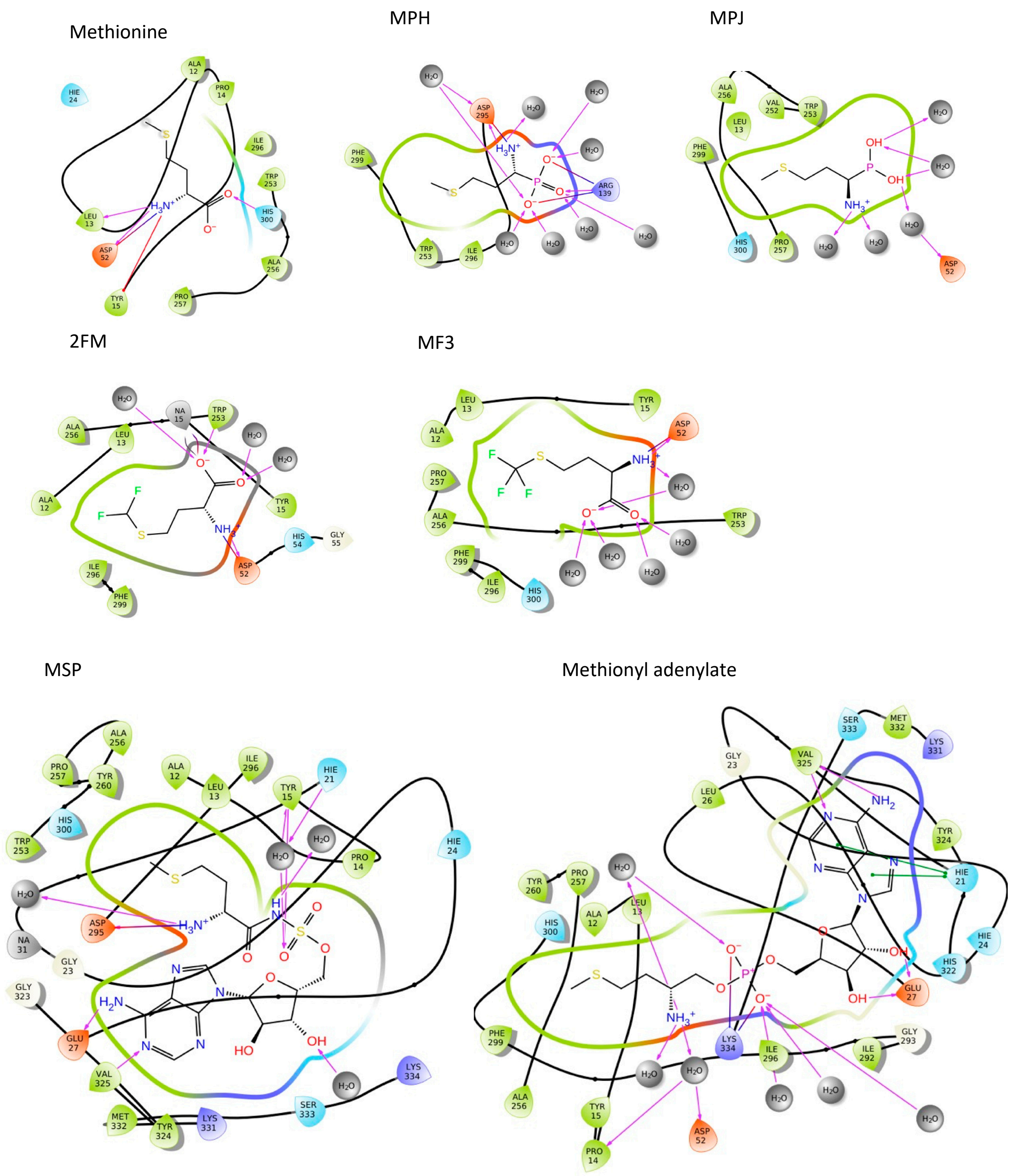

Figure S7. 2D ligand interactions of the final frame of the protein-ligand complexes after 200 ns MD simulation.

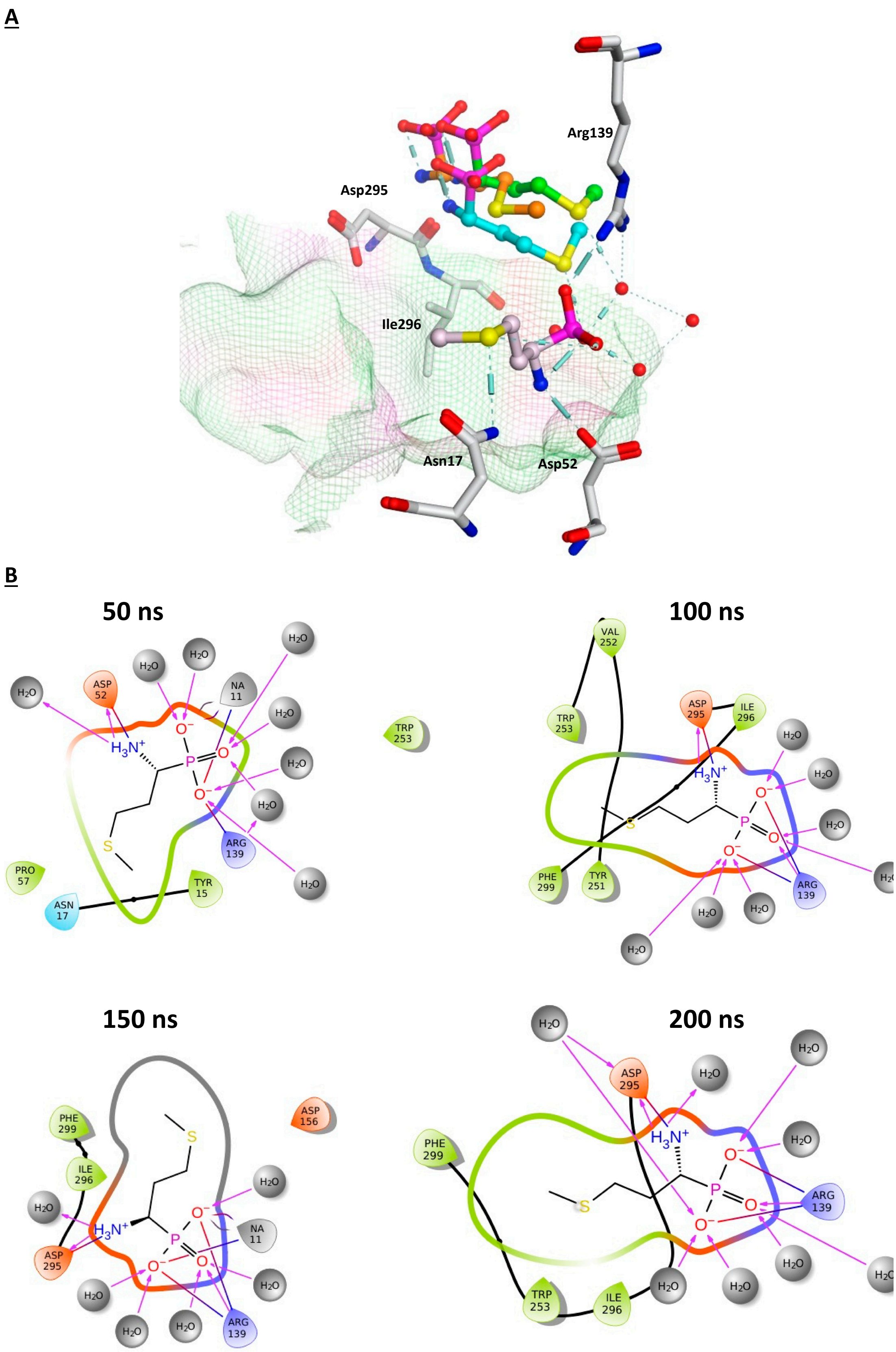

Figure S8. (A) Change in position of MPH over 200 ns MD simulation: 50 ns (light pink), 100 ns (green), 150 ns (orange) and 200 ns (turquoise). (B) 2D ligand interactions of the *P. mirabilis* MetRS-MPH complex at 50, 100, 150 and 200 ns MD simulation.

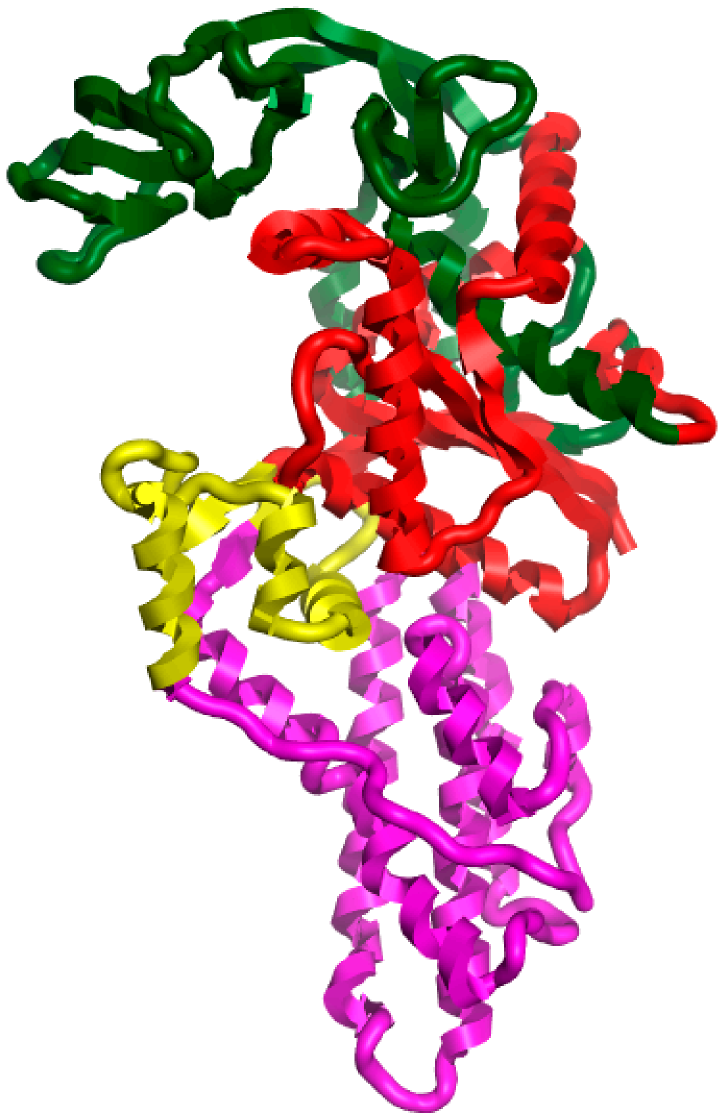

Figure S9: Final *P. mirabilis* MetRS homology model with the characteristic domains in ribbon representation: Rossmann fold: red, connective peptide (CP): green, KMSKS domain: yellow and anticodon domain: purple.
